# Transcriptome Analysis Reveals the Complex Molecular Mechanisms of *Brassica napus*–*Sclerotinia sclerotiorum* Interactions

**DOI:** 10.3389/fpls.2021.716935

**Published:** 2021-10-08

**Authors:** Binjie Xu, Xi Gong, Song Chen, Maolong Hu, Jiefu Zhang, Qi Peng

**Affiliations:** ^1^Key Laboratory of Cotton and Rapeseed, Ministry of Agriculture, Institute of Industrial Crops, Jiangsu Academy of Agricultural Sciences, Nanjing, China; ^2^Innovative Institute of Chinese Medicine and Pharmacy, Chengdu University of Traditional Chinese Medicine, Chengdu, China; ^3^Triticeae Research Institute, Sichuan Agricultural University, Chengdu, China; ^4^Institute of Life Sciences, Jiangsu University, Jiangsu, China

**Keywords:** *Sclerotinia sclerotiorum*, *Brassica napus*, interaction, transcriptome analysis, plant hormones, cell wall enzymes

## Abstract

Sclerotinia stem rot caused by *Sclerotinia sclerotiorum* is a devastating disease for many important crops worldwide, including *Brassica napus*. Although numerous studies have been performed on the gene expression changes in *B. napus* and *S. sclerotiorum*, knowledge regarding the molecular mechanisms of *B. napus*–*S. sclerotiorum* interactions is limited. Here, we revealed the changes in the gene expression and related pathways in both *B. napus* and *S. sclerotiorum* during the sclerotinia stem rot (SSR) infection process using transcriptome analyses. In total, 1,986, 2,217, and 16,079 differentially expressed genes (DEGs) were identified in *B. napus* at 6, 24, and 48 h post-inoculation, respectively, whereas 1,511, 1,208, and 2,051 DEGs, respectively, were identified in *S. sclerotiorum*. The gene ontology and Kyoto Encyclopedia of Genes and Genomes analyses showed that most of the hormone-signaling pathways in *B. napus* were enriched, and thus, the hormone contents at four stages were measured. The DEGs and hormone contents revealed that salicylic acid was activated, while the jasmonic acid pathway was repressed at 24 h post-inoculation. Additionally, the expressional patterns of the cell wall-degrading enzyme-encoding genes in *S. sclerotiorum* and the hydrolytic enzymes in *B. napus* were consistent with the SSR infection process. The results contribute to a better understanding of the interactions between *B. napus* and *S. sclerotiorum* and the development of future preventive measures against SSR.

## Introduction

*Brassica napus* (canola, rapeseed) is the second most widely produced oilseed crop worldwide and is constantly threatened by a devastating disease caused by the fungal pathogen *Sclerotinia sclerotiorum*. *Sclerotinia sclerotiorum* (Lib.) de Bary, the causative agent of sclerotinia stem rot (SSR), is a plant pathogen that belongs to the Sclerotiniaceae family of Ascomycete fungi. It also has a wide host range and can infect more than 400 plant species, including many important crop plants (Boland and Hall, 1994; Kabbage et al., 2015). This fungus is a prototypical necrotrophic pathogen, and it secretes the non-selective phytotoxin oxalic acid (OA), which aids the pathogen in multiple ways, such as pH acidification, Ca^2+^ chelation, and the low-pH activation of degradative enzymes, which augment the fungal colonization of host plants (Xu et al., 2018). Cultivating disease-resistant rapeseed varieties is the most cost-effective way to prevent and control SSR. However, the lack of identified resistance genes in cultivated rapeseed varieties and related species has limited the molecular breeding of rapeseed. Therefore, it is imperative to understand the molecular mechanisms of *B*. *napus* and *S. sclerotiorum* interactions and to create new sources of disease-resistant rapeseed.

Usually, plants respond through two immune pathways when attacked by pathogens: pathogen-associated molecular patterns (PAMP)-triggered immunity and effector-triggered immunity (Jones and Dangl, 2006), which involve physical barriers (e.g., cell walls and a vast array of antimicrobial compounds). Many of these antimicrobial compounds are part of active defense response, and their rapid induction is contingent on the ability of the plant to recognize and respond to invading pathogens (Staskawicz et al., 1995; Baker et al., 1997). Phytohormones, an antimicrobial chemical factor, play crucial roles in plant defenses following a pathogen attack. Generally, plant defense responses against pathogens are controlled by complex signaling pathways that often involve the classical defense phytohormones: salicylic acid (SA), ethylene (ET), and jasmonic acid (JA) (Robert-Seilaniantz et al., 2011). The SA signaling triggers resistance against biotrophic and hemibiotrophic pathogens and the establishment of systemic acquired resistance, whereas a combination of JA and ET signaling activates resistance against necrotrophs (Glazebrook, 2005; Grant and Lamb, 2006).

Because of the availability and efficiency of next-generation sequencing technology, transcriptome analyses have been used to understand the molecular mechanisms of host plant interactions with pathogens. Wu et al. (2016) used transcriptome analysis to classify 13,313 genes according to their functional categories and analyzed the expression levels of genes having hydrolase-related functions. Seifbarghi et al. (2017) used an RNA-sequencing (RNA-seq) analysis to comprehensively catalog genes that are expressed and upregulated during *B. napus* infections, with a particular focus on early events. By examining the global transcriptional changes in *S. sclerotiorum* during the infection of rapeseed plants having different susceptibilities to the pathogen, the roles of peroxisome-related pathways, along with cell-wall degradation and host metabolite detoxification, have been identified (Chittem et al., 2020).

Here, we performed a genome-wide expression profiling of *B. napus* and *S. sclerotiorum* to investigate the defense mechanisms involved in both the resistance of *B. napus* against infections, the *S. sclerotiorum* infection of *B. napus*, and their interactions. For this purpose, we used the rapeseed cultivar “Ning RS-1” that was inoculated with *S. sclerotiorum* isolate 1980 for differential gene expression analyses at three different time points, namely, an early stage of pathogen establishment and the late stages of symptom expression and sporulation. Additionally, hormone contents were measured and analyzed in combination with the expressional patterns of related genes in *B. napus*. An in-depth analysis of differentially expressed genes (DEGs) during infection or in response to *S. sclerotiorum* may provide insights into the molecular mechanisms of the disease resistance of *B. napus*.

## Materials and Methods

### Plant Material, Pathogen, and Pathogen Inoculation

The double haploid *B. napus* cultivar “Ning RS-1,” which shows a partial SSR resistance (Zhang et al., 2002), was used as the host plant. The *S. sclerotiorum* isolate 1980 was used because its genome sequence was available (Derbyshire et al., 2017). The *S. sclerotiorum* was washed with sterilized water and cultured on a potato dextrose agar (PDA) medium (300 g/L of diced potato, 20 g/L of sucrose, and 15 g/L of agar in 1 L of ddH_2_O) for activation at 25°C over 4 days. Then, mycelial plugs (5 mm in diameter) excised from plates with growing fungal cultures were collected and placed on the leaves of 3-month-old rapeseed plants for inoculation. After inoculation, the plants were incubated in a sealed and humidified tray at room temperature. Leaf samples were collected using a 2-cm diameter punch at 6, 24, and 48 h post-inoculation (hpi). The leaves without inoculation and the mycelial plugs were mixed and used as 0 hpi (mock) samples. Three independent biological replicates (three plants/biological replicate/time point) were used for each inoculation experiment. In total, 12 samples were prepared and subjected to RNA-seq analysis.

### RNA Extraction, cDNA Library Construction, and RNA Sequencing

The total RNA was extracted using a TRIzol reagent (TIANGEN, Beijing, China) following the procedure of the manufacturer and checked for quantity and purity with a Bioanalyzer 2100 and RNA 6000 Nano LabChip Kit (Agilent, Santa Clara, CA, USA). Before RNA extraction, the fungi from the culture plates and oilseed leaves at 0 hpi were pooled as a mixed RNA sample. In total, 12 RNA samples (three inoculated samples at 6, 24, and 48 hpi and a mixed mock-fungal sample for each biological replicate) were used for library construction with an TruSeq RNA Sample Preparation kit v2 (Illumina, San Diego, CA, USA) following the instructions of the manufacturer. All the samples were sequenced using an Illumina HiSeq 2000 sequencer by Biomac Inc. (Beijing, China).

### Data Processing, Read Mapping, and Differential Gene Expression Analysis

Various quality controls for raw reads were conducted using FastQC version 0.1.9 (Brown et al., 2017) to remove the primer/adaptor sequence-containing and low-quality [in which the number of bases with PHRED-like scores (Q-score) of <20 exceeded 30%] reads. Then, the first 10 bp of the reads that showed unstable base compositions as determined by the percentages of four different nucleotides (A, T, C, and G) and the low-quality bases (Q-score <20) from the 3′ ends of the reads were trimmed, and the reads of <50 bp were removed. All the high-quality reads of each sample that passed the quality control assays were mapped independently to the *B. napus* and *S. sclerotiorum* genomes using TopHat 2 (Trapnell et al., 2012; Kim et al., 2013) with the default parameters. The reference genomes of *B. napus* were downloaded from http://www.genoscope.cns.fr/brassicanapus/data/, whereas those of *S. sclerotiorum* were downloaded from the National Center for Biotechnology Information search database (https://www.ncbi.nlm.nih.gov/assembly/GCA_001857865.1/). Only uniquely mapped reads were used for further gene expression analyses. For all the comparisons, read counts were normalized to the aligned fragments per kilobase of transcript per million (FPKM) mapped reads (Mortazavi et al., 2008) to obtain the relative expression levels. Differential expression analyses between different developmental stages were performed using the DESeq R packages (Wang et al., 2010). The DEGs between different samples were identified using the restrictive conditions of an absolute value of fold change ≥4 and a false discovery rate ≤ 0.001.

### Gene Ontology (GO) and Kyoto Encyclopedia of Genes and Genomes (KEGG) Enrichment Analyses

For the gene functional annotation, all the genes in *B. napus* and *S. sclerotiorum* were used as queries against the National Center for Biotechnology Information non-redundant protein (https://blast.ncbi.nlm.nih.gov/Blast.cgi), Swiss-Prot (Apweiler et al., 2004), and Pfam (Finn et al., 2014) databases. The GO terms associated with each BLAST hit were annotated using Blast2GO (Conesa et al., 2005). Then, all the *B. napus* and *S. sclerotiorum* genes were used as queries against the InterPro database (http://www.ebi.ac.uk/interpro/) using InterProScan550 (Jones et al., 2014). Finally, the GO terms of the *B. napus* and *S. sclerotiorum* genes were annotated by merging the Blast2GO and InterPro annotation results. The GO enrichment analysis provided all the GO terms that were significantly enriched with DEGs compared with the genome background using Blast2GO with a false discovery rate of ≤ 0.01. The annotations were then refined and enriched using the TopGo R package (http://www.bioconductor.org/packages/release/bioc/html/topGO.html). The enrichment of DEGs in the Kyoto Encyclopedia of Genes and Genomes (KEGG) pathways was analyzed using the KOBAS software 2.0 (Xie et al., 2011). The heat maps were drawn using the R package and TBtools (Chen et al., 2020) based on the log_2_ transformed FPKM values. The expression value for a given gene was normalized.

### Quantitative RT-PCR Assays

Quantitative real-time-PCR assays were performed to confirm the RNA-seq results and analyze the expression level of target genes. In total, 2 μg of the total RNA from each sample (the same samples used for RNA-seq) were used to synthesize cDNA with a TransScript One-Step gDNA Remover and cDNA Synthesis Kit following the instructions of the manufacturer (TaKaRa, Dalian, China). The quantitative real-time-PCR (qRT-PCR) was performed using an SYBR premix Ex Taq™ RT-PCR kit (TaKaRa). All the experiments were performed following the instructions of the manufacturer. The data were collected from three biological and three technical replicates. The transcript level was normalized using three reference genes, *Actin 2* (BnaC03g73810D), *Ubiquitin-conjugating enzyme 10* (BnaA10g06670D), and *Yellow Leaf Specific 8* (BnaC09g47620D). The primers used in these experiments are listed in Supplementary Table 1.

### Measurement of Hormones in *B. napus* Leaves

The samples infected with *S. sclerotiorum* were prepared and collected in the same manner described for the RNA-seq analysis. In addition, samples taken from uninfected areas of the same leaves were collected. The SA, JA, gibberellic acid (GA_3_), indole-3-acetic acid (IAA), and abscisic acid (ABA) concentrations were measured using an ultraperformance liquid chromatography-tandem mass spectrometry (UPLC-MS/MS) method (Balcke et al., 2012) with minor modifications. The phytochemical standards were purchased from Sigma-Aldrich (St. Louis, MO, USA), whereas other reagents were purchased from Solarbio (Beijing, China). Three biological replicates per hormone were analyzed.

## Results

### A DEG Analysis of *B. napus* Infected Leaves Using RNA-seq

**Figure 1:**
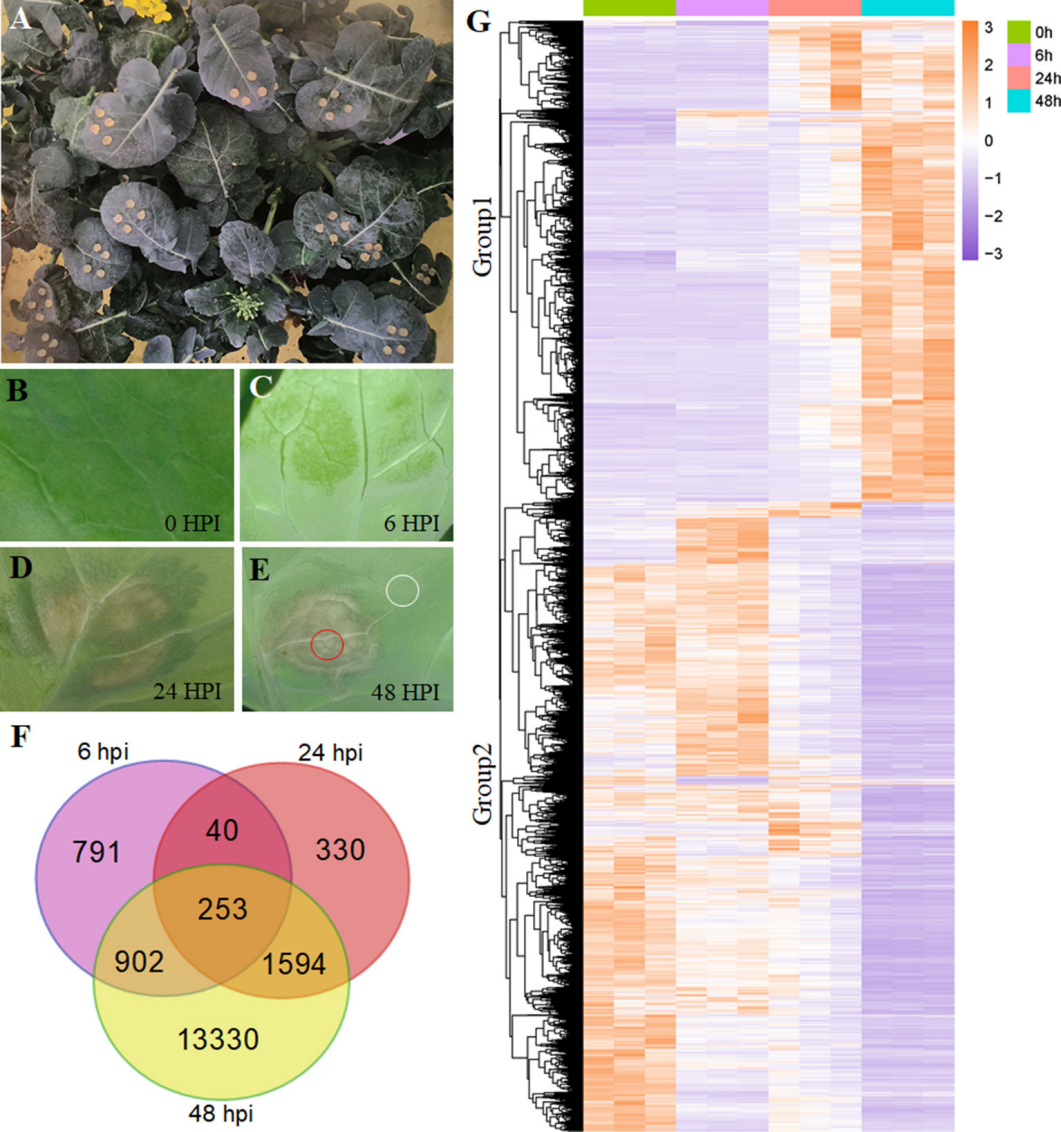
Differentially expressed gene (DEG) analysis in *Brassica napus*. **(A)** The growing plant of *B. napus* at the flowering stage. **(B–E)** The symptoms of *Sclerotinia sclerotiorum* infection on *B. napus* leaves at 0, 6, 24, and 48 hpi. The circled areas in **(E)** were used in **Figure 5**. **(F)** Overlapping and unique DEGs at the 6-, 24-, and 48-hpi stages. **(G)** Heatmap illustrating the hierarchical clustering results for RNA-sequencing (RNA-seq), group 1 and group 2 represent two expressed patterns. hpi, hours post-inoculation.

*Sclerotinia sclerotiorum* was inoculated into the *B. napus* leaves at the early flowering stage (Figure 1A). The leaf necrosis symptom was not observed at 0 and 6 hpi, but it was significant at 24 hpi, and the *S. sclerotiorum* rapidly infected the *B. napus* leaves between 24 and 48 hpi (Figures 1B–E). The RNA-seq data revealed that approximately 44.7 to 66.4 million paired-end reads were generated in *B. napus* (Supplementary Table 2). After mapping to the reference genome using TopHat2 (v2.0.12) and merging annotations through cuffmerge, 102,216 genes were annotated to the published reference genome and 2,112 new genes were annotated and predicted (Supplementary Table 3).

Using the gene expression levels calculated by FPKM mapped reads, we found a tight overlap among the three stages (6, 24, and 48 hpi) compared with the mock stage (0 hpi; Figure 1F), having 253 DEGs. The numbers of genes expressed in only one stage were 791, 330, and 13,330 for 6, 24, and 48 hpi, respectively. At the 48-hpi stage, the highest numbers of DEGs (16,079) and stage-specific genes (13,330) were identified, indicating that time was required to genetically respond to the *S. sclerotiorum* infection of *B. napus*.

### Functional Classification Using GO and KEGG Pathway Analyses in *B. napus*

**Figure 2:**
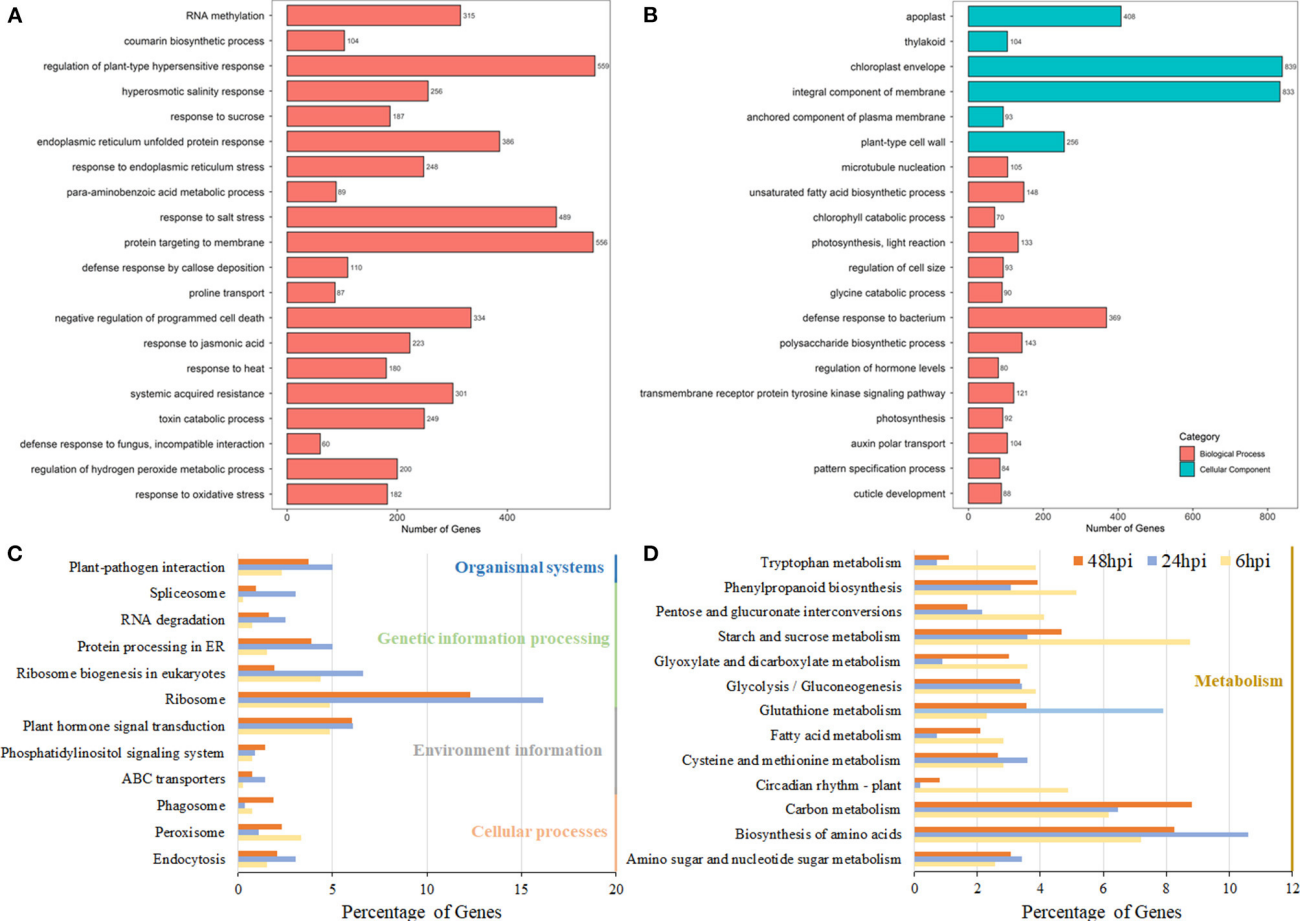
Pathway analysis of DEGs in *B. napus* based on the gene ontology (GO) and Kyoto Encyclopedia of Genes and Genome (KEGG) databases. The GO enrichment analysis of Group 1 **(A)** and Group 2 **(B)**. The y-axis indicated the numbers of annotated genes, and the x-axis indicated the GO terms. The GO analysis was conducted using the Blast2Go software. **(C,D)** The KEGG enrichment analysis of DEGs at the 6-, 24-, and 48-hpi stages. The x-axis indicated the ratio of the enriched gene number to the total gene number at three stages, and the y-axis indicated different enriched terms.

A hierarchical clustering analysis used to compare global gene expression changes showed two significantly different expression pattern groups (Figure 1G). Furthermore, the GO analysis indicated that these upregulated DEGs during pathogen infection (8,602 genes clustered in Group 1) were enriched in 20 subcategories of biological processes, including the regulation of plant-type hypersensitive response, protein targeting to the membrane, responses to stress processes, the negative regulation of programmed cell death (PCD), systemic acquired resistance, and responses to JA (Figure 2A; Supplementary Table 4), whereas the downregulated DEGs (11,164 genes clustered in Group 2) were enriched in both cellular components and biological processes, including the chloroplast envelope, an integral component of the membrane, plant-type cell wall, auxin polar transport, and the regulation of hormone levels (Figure 2B; Supplementary Table 4).

**Figure 3:**
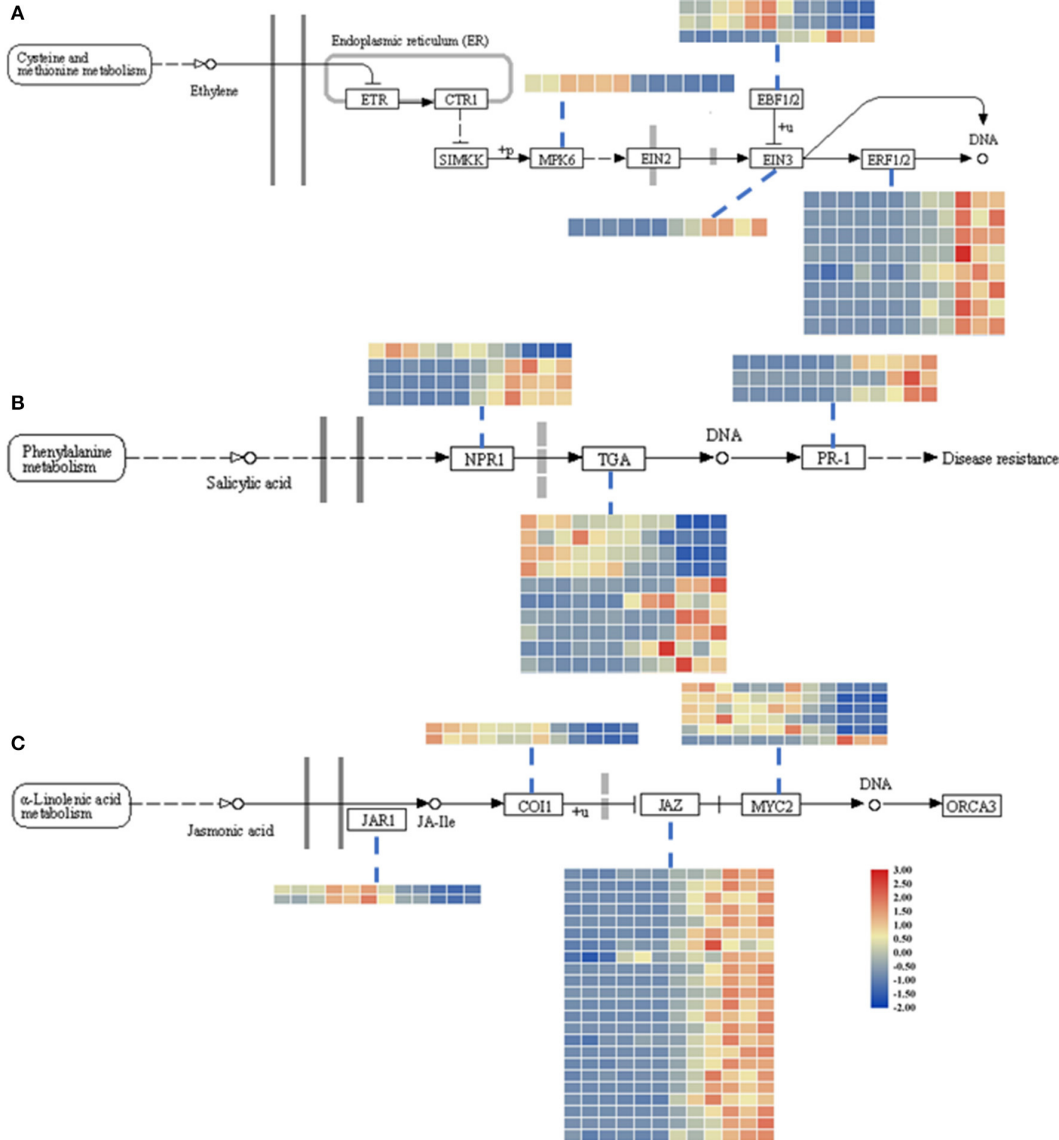
Heatmap of DEGs related to the ET (ethylene; **A**), SA (salicylic acid; **B**), and JA (jasmonic acid; **C**) signaling pathways. The descriptions of DEGs in this picture were listed in Table 1. The heatmap was drawn with TBtools (Chen et al., 2020).

Using all the DEGs at the three developmental stages, 6, 24, and 48 hpi, compared with the mock stage, 389, 557, and 3,946 genes were mapped to the KEGG database, respectively. At 24 and 48 hpi, these DEGs were classified into 50 different terms, with the ribosome (90/557 and 485/3,946, respectively) and the biosynthesis of amino acids pathway (59/557 and 326/3,946, respectively) accounting for large proportions of the DEGs (Figures 2C,D; Supplementary Table 5). Most DEGs were classified into pathways related to starch and sucrose metabolism (34/389) and the biosynthesis of amino acids (28/389) at the 6-hpi stage (Figure 2D). During the infection, the number of DEGs in plant-pathogen interactions and plant hormone signal transduction pathways increased dramatically (Figure 2C). A detailed analysis showed that the DEGs grouped into the plant hormone signal transduction pathway category were mainly classified as being involved in JA and ET signal transduction and brassinosteroid biosynthesis (Supplementary Table 6). At 6 hpi, DEGs were classified into the auxin/IAA, cytokinin, ABA, and JA pathways (Figure 3; Table 1; Supplementary Table 6), which indicated that these hormone signals were activated at the early stage of pathogen infection. Then, more hormone signal pathways, including that of SA, were activated, consistent with the pathogen invasion (Figures 3B,C).

**Table 1:** Description and expression of genes in *B. napus* involved in the hormone signaling pathway.

**Gene ID**	**Description^a^**	**Expression level (hpi)^b^**		
		**6-**	**24-**	**48-**
BnaA04g01520D	Mitogen-activated protein kinase 6	–	−2.88	−6.01
BnaA09g04600D	EIN3-binding F-box protein 2	–	–	−1.95
BnaC09g04050D	EIN3-binding F-box protein 2	–	–	−2.29
BnaC07g29410D	EIN3-binding F-box protein 2	–	1.64	2.27
BnaC06g23450D	Ethylene insensitive 3	–	2.22	2.77
BnaA01g23940D	Ethylene-responsive transcription factor	−2.06	4.26	5.93
BnaA04g28820D	Ethylene-responsive transcription factor	–	>100	>100
BnaA09g50010D	Ethylene-responsive transcription factor	–	5.62	8.16
BnaA10g04090D	Ethylene-responsive transcription factor	–	3.44	6.02
BnaC03g17400D	Ethylene-responsive transcription factor	–	1.91	2.58
BnaC04g42060D	Ethylene-responsive transcription factor	1.52	2.59	4.91
BnaC05g04210D	Ethylene-responsive transcription factor	–	5.65	7.41
BnaC08g44670D	Ethylene-responsive transcription factor	–	5.47	7.65
BnaA09g11550D	Regulatory protein NPR1	–	–	−4.05
BnaA01g15310D	Regulatory protein NPR2	–	2.02	2.70
BnaAnng05410D	Regulatory protein NPR3	–	3.74	4.18
BnaC07g02890D	Regulatory protein NPR3	–	3.29	3.28
BnaA02g00310D	Transcription factor TGA4	–	–	−2.91
BnaA07g33790D	Transcription factor TGA7	–	–	−2.15
BnaC06g20630D	Transcription factor TGA7	–	–	−3.45
BnaC09g46670D	Transcription factor TGA4	–	−1.40	−4.00
BnaA06g04770D	TGACG-sequence-specific DNA-binding protein TGA-2.1	–	–	3.42
BnaA08g20970D	Transcription factor TGA3	–	4.62	3.88
BnaA09g48940D	TGACG-sequence-specific DNA-binding protein TGA-2.1	–	−1.44	3.42
BnaC05g06030D	TGACG-sequence-specific DNA-binding protein TGA-2.1	−1.33	−0.88	2.89
BnaC08g20170D	Transcription factor TGA3	1.87	4.54	3.61
BnaUnng03970D	Transcription factor HBP-1b	−3.82	1.52	3.40
BnaA03g38630D	Pathogenesis-related protein 1	–	6.00	6.63
BnaC01g04530D	Pathogenesis-related protein 1	−1.17	2.57	10.04
BnaC03g45470D	Pathogenesis-related protein 1	–	3.49	4.88
BnaA05g01450D	Jasmonic acid-amido synthetase JAR1	–	–	−2.76
BnaC04g01170D	Jasmonic acid-amido synthetase JAR1	1.21	–	−1.91
BnaA05g05650D	Coronatine-insensitive protein 1	–	–	−2.40
BnaC04g05430D	Coronatine-insensitive protein 1	–	–	−3.48
BnaA02g05120D	Protein TIFY 3B	–	1.84	3.31
BnaA02g15990D	Protein TIFY 11B	–	3.76	4.66
BnaA03g04250D	Protein TIFY 9	–	>100	>100
BnaA05g22360D	Protein TIFY 6B	–	2.66	3.32
BnaA06g13250D	Protein TIFY 10A	–	6.18	7.61
BnaA07g23750D	Protein TIFY 7	–	4.67	4.83
BnaA07g30200D	Protein TIFY 11B	2.43	4.64	4.00
BnaA07g31880D	Protein TIFY 10B	1.60	1.56	2.25
BnaA08g22180D	Protein TIFY 10A	–	4.90	6.55
BnaA08g23150D	Protein TIFY 11A	–	5.16	6.28
BnaA10g20060D	Protein TIFY 9	–	6.39	8.78
BnaC02g45660D	Protein TIFY 11B	–	3.34	4.34
BnaC03g71460D	Protein TIFY 9	–	7.73	8.29
BnaC05g14810D	Protein TIFY 10A	–	5.92	7.66
BnaC05g35610D	Protein TIFY 6B	–	1.58	2.35
BnaC06g24560D	Protein TIFY 7	–	5.34	6.50
BnaC06g33640D	Protein TIFY 11B	–	3.09	4.14
BnaC08g18640D	Protein TIFY 10A	–	3.24	3.66
BnaC08g36840D	Protein TIFY 10A	–	6.57	7.32
BnaC08g37780D	Protein TIFY 11A	–	2.47	2.38
BnaC08g48340D	Protein TIFY 11A	–	6.38	7.62
BnaC09g43860D	Protein TIFY 9	−1.09	5.16	6.96
BnaCnng08230D	Protein TIFY 10B	–	1.43	2.27
BnaA01g08750D	Transcription factor MYC2	−1.80	–	−4.39
BnaA09g18200D	Transcription factor MYC2	–	–	−4.26
BnaC01g10420D	Transcription factor MYC2	–	–	−4.03
BnaC07g19530D	Transcription factor MYC2	–	−2.13	−5.81
BnaC09g19710D	Transcription factor MYC2	–	–	−3.69
BnaA09g18160D	Transcription factor MYC2	–	–	2.28

a *Annotation based on the presence of conserved Pfam domains and the BLAST report*.

b *Expression change (Log_2_FC) relative to 0 h post-inoculation (hpi). (–) No significant change in expression*.

*More information about the genes can be found in Supplementary Table 3*.

### Expression Patterns of Defense Response-Associated Genes

**Figure 4:**
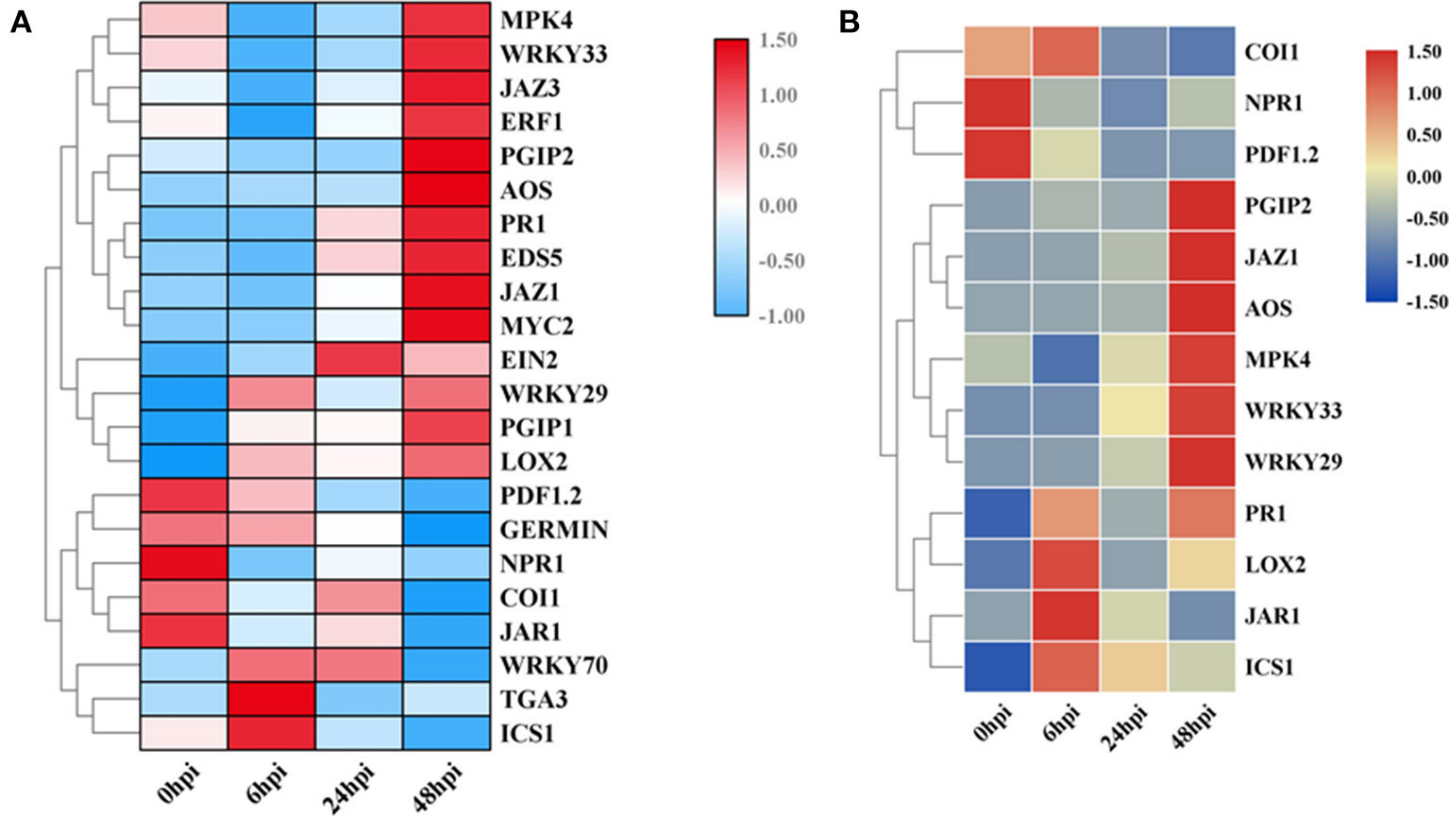
Validation of the expression of 22 genes by quantitative reverse transcription PCR (qRT-PCR). **(A)** The expression-level changes based on qRT-PCR data. **(B)** The expression-level changes based on RNA-seq data. Gene IDs are listed in Supplementary Table 1.

The RNA-seq data were verified through a qRT-PCR analysis of eight DEGs at four stages (Supplementary Table 1). The expression patterns of the eight genes as determined by the qRT-PCR were largely consistent with those obtained from RNA-seq (Figure 4).

Additionally, to identify the expression patterns of genes involved in defense-response pathways, which play crucial roles in a pathogen attack, several genes were selected and analyzed by qRT-PCR (Figure 4A). In the JA pathway, the expression levels of two genes involved in JA biosynthesis at four stages were identified. The expression levels of *allene oxide synthases* (*AOS*; BnaC02g29610D) and *lipoxygenase 2* (*LOX2*; BnaA07g19600D), two genes encoding the key enzymes of JA biosynthesis, increased as the pathogen infection proceeded, while the expression levels of *JAR1*, coronatine-insensitive 1 (*COI1*), and *MYC2* were more complicated (Figure 3C). In particular, the expression levels of jasmonate ZIM-domain proteins (JAZs), the key proteins involved in the JA-signaling pathway, in binding to *COI1* through Skp1/Cullin1/F-box protein COI1 (SCF^coi1^) complex-mediated ubiquitination and in regulating ubiquitin-26S proteasome degradation, were downregulated (Figure 3C). In rapeseed, the mitogen-activated protein kinase cascade reaction and its direct targets, *WRKY* transcription factors, play broad roles in regulating defenses (Eulgem and Somssich, 2007). The expression levels of *MPK4, WRKY33, WRKY29*, and *WRKY70* were analyzed by qPCR, and these genes were all upregulated after 6 hpi (Figure 4). *Isochorismate synthase 1* (*ICS1*) is an important gene involved in SA biosynthesis (Zheng et al., 2015). In this study, the expression level of *BnICS1* (BnaC06g22820D) showed a significant increase at 6 hpi and then decreased at 24 and 48 hpi (Figure 4A). Polygalacturonase-inhibiting proteins (PGIPs) occur in plant cell walls and counteract the actions of the polygalacturonase (PG) from *S. sclerotiorum* to prevent the degradation of cell walls (De Lorenzo and Ferrari, 2002). The expression levels of *PGIP1* and *PGIP2* were upregulated consistently after inoculation and peaked at 48 hpi (Figure 4A).

### Several Hormone Contents Changed in *B. napus* Leaves After *S. sclerotiorum* Infection

**Figure 5:**
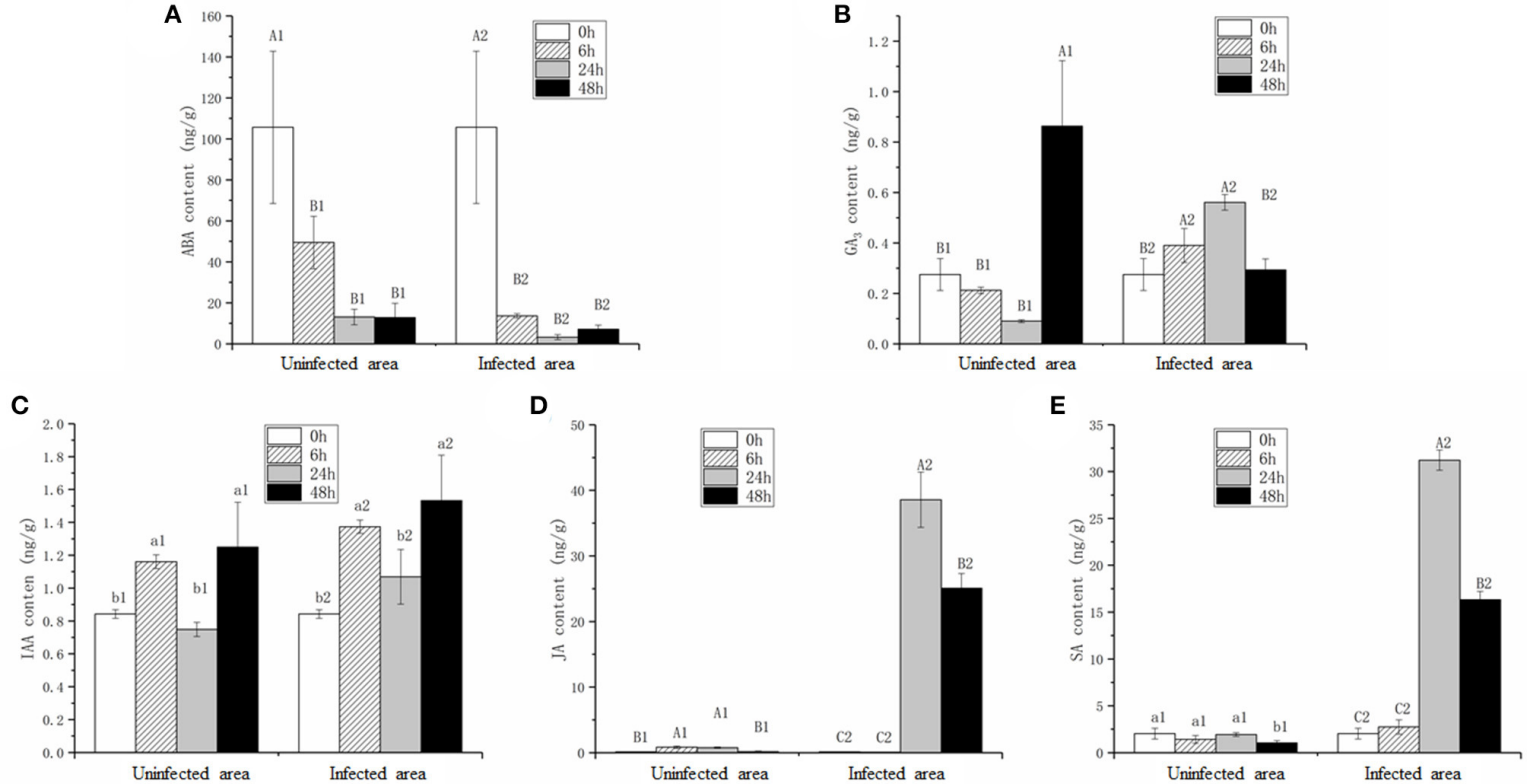
Contents of abscisic acid (ABA) **(A)**, gibberellic acid (GA3) **(B)**, indole-3-acetic acid (IAA) **(C)**, JA **(D)**, and SA **(E)** in infected (infected area) and uninfected leaves (uninfected area). Values are the mean ± SD of three biological replicates per treatment. Different letters above each column indicate a significant difference (Capital letters: *p* < 0.01; others: *p* < 0.05; *n* = 3).

Considering the differential expression of genes involved in plant hormone pathways, the contents of five hormones, ABA, IAA, GA_3_, JA, and SA, were measured (Figure 5). To distinguish the hormonal differences in infected and uninfected areas (white circle in Figure 1E), the hormone contents at four stages were measured. It was hypothesized that the trends in the hormone levels in the uninfected leaves were related to plant resistance, whereas the hormone levels in the infected leaves were related to plant-pathogen interactions. The ABA content in the infected leaves was reduced significantly (*p* < 0.01) during pathogen infection (Figure 5A), while the GA_3_ content was maintained at the same level at 0 and 48 hpi after significantly increasing at 6 and 24 hpi (Figure 5B). The IAA content at 48 hpi was greater than in the mock stage (*p* < 0.05; Figure 5C). The JA and SA contents were stable at 6 hpi, increased significantly (*p* < 0.01) at 24 hpi, and then decreased slightly at 48 hpi (Figures 5D,E).

The ABA and IAA contents in uninfected leaves were consistent within the infected leaves (Figures 5A,C), whereas the JA and SA contents in the uninfected leaves were extremely lower compared with the infected leaves at 24 and 48 hpi (Figures 5D,E). Interestingly, the GA_3_ content change trends in the uninfected and infected leaves during pathogen infection were opposite (Figure 5B).

### Differential Gene Expression Analysis in *S. sclerotiorum*

**Figure 6:**
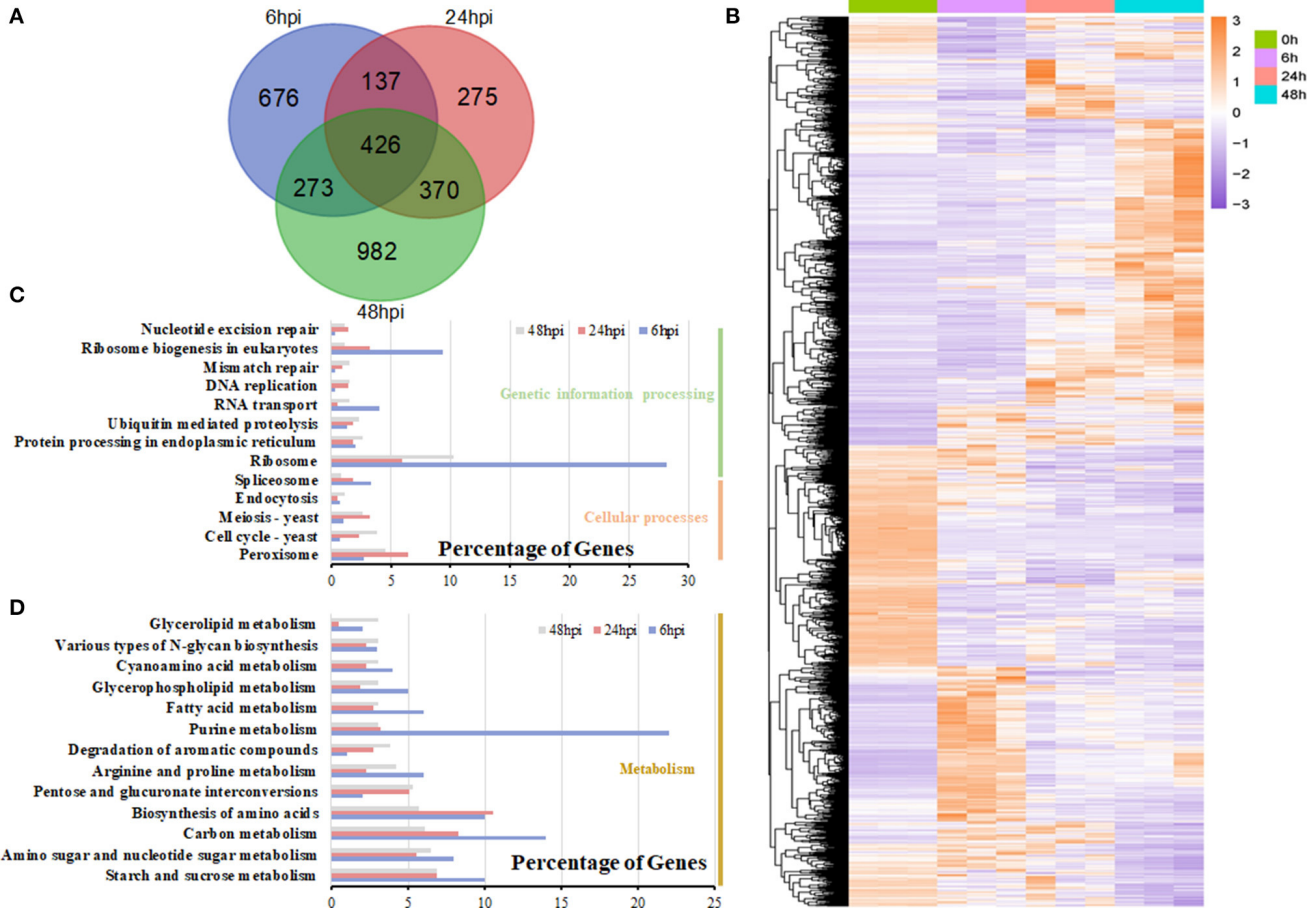
DEG analysis in *S. sclerotiorum*. **(A)** The number of DEGs expressed at the 6-, 24-, and 48-hpi stages and their overlapping. **(B)** Heatmap illustrating the hierarchical clustering results for RNA-seq. **(C,D)** KEGG enrichment analysis of DEGs at the 6-, 24-, and 48-hpi stages. The x-axis indicated the ratio of the enriched gene number to the total gene number at three stages, and the y-axis indicated different enriched terms.

During the infection, many expressed genes of *S. sclerotiorum* were induced and changed, with the peak occurring at 48 hpi (Figure 6A; Supplementary Table 7). The expression patterns of *S. sclerotiorum* genes varied and clustered into different sub-clusters (Figure 6B). Some DEGs were enriched in cell-wall modification (GO:0005618 and GO:0042545) and the chitin/chitinase process (GO:0006032 and GO:0004568), which play important roles during host invasion. The KEGG analysis showed that the DEGs of *S. sclerotiorum* at 6 hpi were mainly enriched in the ribosome and its biogenesis pathway (Figure 6C). Then, at 24 hpi, the DEGs were enriched in the valine, leucine and isoleucine degradation, pentose and glucuronate interconversions, starch and sucrose metabolism, and peroxisome pathways (Figures 6C,D). At 48 hpi, some DEGs showed enrichment in the pentose and glucuronate interconversions, starch and sucrose metabolism, and alpha-linolenic acids pathway (Figures 6C,D; Supplementary Table 8), in which the expression levels of shikimate dehydrogenase (*SS1G_08336*; gene 7815), chorismate mutase type II (*SS1G_08569*; gene 8012), and galactose oxidase (*SS1G_13392*; gene10461) were upregulated. Many genes in these pathways, including *SS1G_00468, SSPG1, SS1G_07184*, and *SS1G_01021*, were upregulated (Supplementary Table 9).

**Figure 7:**
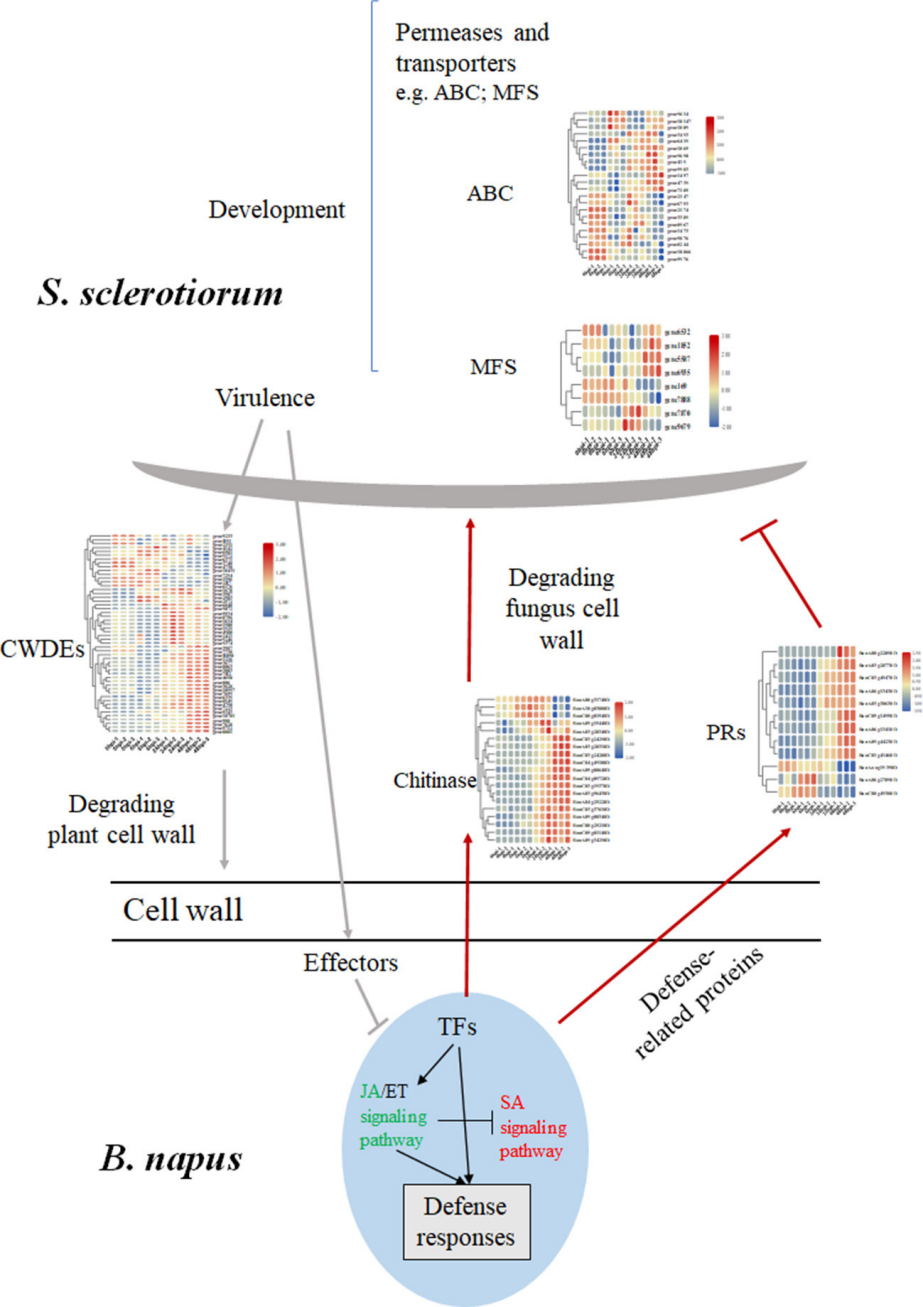
Differential changed genes involved in the major molecular mechanisms of *B. napus*–*S. sclerotiorum* interactions. The SA signaling pathway colored in red indicated an activation, while the JA signaling pathway colored in green indicated repression in our study. Abbreviations: CWDE, cell wall-degrading enzymes; PRs, pathogen-related proteins; TF, transcription factor.

The sclerotium is central to the life and disease cycles of *S. sclerotiorum* (Bolton et al., 2006), which require the expression of numerous associated genes. As a group, the ATP-binding cassette and major facilitator superfamily (MFS) transporters exhibit wide ranges of specificities (amino acids, drugs, heavy metals, inorganic ions, peptides, polysaccharides, and sugars); however, some have been implicated in the secretion of fungal toxins or the efflux of host phytoalexins (Perlin et al., 2014). Here, 8 genes encoding MFS transporters and 22 genes encoding ATP-binding cassette transporters were detected and showed differential expression levels during the infection stage (Figure 7). Interestingly, almost all the differential expressed *MFS* genes encoded sugar transport proteins but showed various expression patterns (Figure 7; Supplementary Table 9). For instance, the expression level of *SS1G_05572* (gene 6935) was upregulated at 48 hpi, while *SS1G_08425* (gene 7888) was significantly downregulated compared with 0 hpi, which suggested unknown and diverse functions for these *MFS* genes (Figure 7).

Numerous genes encoding enzymes with hydrolytic activities were induced during infection, with the largest group encoding carbohydrate-active enzymes. Most of the predicted carbohydrate-active enzyme-encoding genes were from the glycoside hydrolase (GH) and carbohydrate esterase (CE) families, and they were upregulated during the *S. sclerotiorum* infection process (Amselem et al., 2011; Seifbarghi et al., 2017). In this research, 52 genes belonging to the GH family were identified, and most showed increased expression levels during the infection (Table 2). Among them, the GH28 subfamily contains PGs, enzymes that degrade cell-wall pectin. *SsPG1* (Gene11050), *SsPG5* (Gene1929), and *SsPG6* (Gene9072), which encode PG and endopolygalacturonase (endo-PG), respectively, were upregulated at 24–48 hpi (Figure 7; Table 2).

**Table 2:** Description and expression of genes in *S. sclerotiorum* involved in CWDEs.

**ID**	**Gene ID**	**Description^a^**	**Expression level (hpi)^b^**		
			**6-**	**24-**	**48-**
1. Glycosyl hydrolases family					
1.1 Carbohydrate transport and metabolism					
Gene4730	SS1G_05915	Function unknown	−2.76	−3.65	−5.59
Gene6176	SS1G_11499	Function unknown	−0.65	−3.32	−3.29
Gene6581	gene6581	Function unknown	1.86	0.58	−2.46
Gene10781	SS1G_09367	Alpha/beta-glucosidase	0.24	0.54	2.29
Gene1191	SS1G_01083	Alpha-glucosidase	−0.89	−0.37	−0.26
Gene1956	SS1G_04148	Alpha-mannosidase	−1.50	−1.14	0.21
Gene9373	SS1G_11763	Beta-galactosidase	0.27	1.62	1.65
Gene4690	SS1G_05977	Beta-mannosidase B	−0.69	0.81	2.36
Gene10997	SS1G_10092	Endo-1,4-beta-xylanase 11A	−1.44	1.01	3.32
Gene8949	SS1G_11212	Endochitinase 33	0.26	1.95	3.15
Gene2117	SS1G_13155	Endochitinase A	0.34	−3.92	−5.32
Gene6339	SS1G_11700	Endochitinase B1	2.07	3.46	4.69
Gene8881	SS1G_11304	Endochitinase B1	0.18	0.89	2.20
Gene11050	SS1G_10167	Endo-polygalacturonase	−2.18	1.54	2.40
Gene1634	BC1G_11909	Endo-xylogalacturonan hydrolase A	0.38	4.00	7.57
Gene1908	SS1G_04207	Exopolygalacturonase X-1	–	5.52	8.34
Gene7294	SS1G_10617	Glucoamylase	1.08	−1.49	−1.32
Gene7659	SS1G_08118	Glucosidase 2 subunit alpha	−0.22	1.05	2.51
Gene5233	SS1G_07184	Invertase 2	−0.73	−0.98	−0.67
Gene2572	SS1G_00677	Killer toxin subunit beta	1.94	0.90	0.45
Gene5534	SS1G_12510	Killer toxin subunit beta	–	0.09	−1.09
Gene2503	SS1G_00773	Killer toxin subunit beta	4.20	2.19	2.22
Gene6840	SS1G_05454	Killer toxin subunit beta	0.49	2.53	1.58
Gene9072	SSPG6	Polygalacturonase	−0.23	1.52	1.62
Gene2421	SS1G_00892	1,4-beta-D-glucancellobiohydrolase C	0.03	1.28	4.11
Gene2331	SS1G_01005	Alpha/beta-glucosidase	−1.37	−0.93	−0.68
Gene9246	SS1G_11922	Arabinanendo-1,5-alpha-L-arabin- osidase A	−0.40	1.71	3.32
Gene7628	SS1G_03647	Beta-galactosidase A	0.32	0.74	2.23
Gene3803	SS1G_02781	Beta-galactosidase B	−0.11	1.97	4.05
Gene7112	SS1G_10842	Beta-galactosidase C	0.18	3.17	4.69
Gene806	SS1G_01572	Beta-galactosidase E	−1.74	0.86	2.70
Gene7223	SS1G_10698	Endopolygalacturonase D	0.33	5.96	7.04
Gene4142	SS1G_12057	Exopolygalacturonase C	1.06	0.38	−0.26
Gene8756	SS1G_14449	Exopolygalacturonase C	−1.60	1.64	2.66
Gene4790	SS1G_05832	Exopolygalacturonase X	0.96	5.39	5.64
Gene6371	SS1G_04852	Glucan endo-1,3-beta-glucosidase	−0.37	−0.25	−1.90
Gene1216	SS1G_12930	Glucan endo-1,3-beta-glucosidase	−0.24	1.71	2.53
Gene5343	SS1G_07039	Rhamnogalacturonase B	1.98	1.82	4.10
Gene3507	SS1G_02399	Rhamnogalacturonase B	0.92	1.62	1.38
Gene8063	SS1G_08634	Galacturan1,4-alpha-galactur- onidase C	−1.21	2.06	4.44
Gene7738	SS1G_08229	Rhamnogalacturonase A	−2.79	−1.85	0.77
1.2 Cell wall/membrane/envelope biogenesis					
Gene1929	polygalacturonase 5	Endopolygalacturonase E	–	4.96	6.20
1.3 general function prediction only					
Gene5712	SS1G_03540	Alpha-L-rhamnosidase rgxb	−0.04	0.89	2.94
Gene3628	gene3628	Alpha-L-rhamnosidase rgxb	−0.34	2.17	4.73
Gene588	SS1G_01833	Arabinan endo-1,5-alpha-L-arabinosidase B	0.39	0.64	3.02
Gene9172	SS1G_12024	Cell surface mannoprotein	0.74	0.35	−0.91
Gene10431	SS1G_09118	Endo-1,3(4)-beta-glucanase	4.42	2.46	6.39
Gene1678	SS1G_04497	Glycosidase crf1	0.48	1.10	0.62
Gene3587	SS1G_02501	Glycosidase crf2	−0.98	−0.39	0.81

a *Annotation based on the presence of conserved Pfam domains, SWISS, and the BLAST report*.

b *Expression change (Log_2_FC) relative to mock. (–) No significant change in expression*.

*More information about the genes can be found in Supplementary Table 9*.

## Discussion

When rapeseeds are attacked by *S. sclerotiorum*, the response involves a range of physiological and biochemical activities. First, rapeseeds form protective barriers, which involve enhanced lignin monomer production, to prevent infection and fungal expansion (Uloth et al., 2016). Then, *S. sclerotiorum* may be killed by different active antimicrobial products formed by rapeseeds, such as indole glycosides, or by the production of chitinase and β-1, 3-glucanase, which degrade the cell walls of *S. sclerotiorum* (Stotz et al., 2011; Zhang et al., 2015). Third, enzymes or chemicals are formed to inhibit the virulence factors of *S. sclerotiorum*, such as PGIP, which is produced by rapeseeds to inhibit the plant cell wall-degrading enzyme PG secreted by *S. sclerotiorum* (De Lorenzo and Ferrari, 2002). Additionally, it is necessary for *S. sclerotiorum* to evolve mechanisms that encourage infection. Usually, pathogenic factors, like OA, are secreted to affect signal transduction in host cells (Kabbage et al., 2015). *Sclerotinia sclerotiorum* promotes cell death by inducing SA synthesis in the host, and numerous effectors secreted by *S. sclerotiorum* participate in host-pathogen interactions (Amselem et al., 2011; Derbyshire et al., 2017).

### Phytohormones and Their Signaling Pathways Play Different Roles in *B. napus* Defense Responses

The role of plant hormones in plant defenses against pathogens, especially JA, SA, and ET, has been well studied (Bari and Jones, 2009). In this research, the contents of five hormones were detected, and the changing trends of IAA and ABA in infected and uninfected leaves were similar (Figures 5A,C), indicating that the two hormones were not directly involved in *B. napus–S. sclerotiorum* interactions. An important plant growth and development regulator, GA_3_, belongs to the gibberellins family and is known to stimulate diverse aspects of developmental processes (Hedden and Phillips, 2000; Yamaguchi, 2008; Sun, 2011). Previously, the role of GA in the signaling involved in defense responses received little attention. However, GA signaling components, especially the negative regulator DELLA, play major roles in plant disease resistance and susceptibility by modulating JA- and SA-dependent responses (Achard et al., 2006, 2008; Navarro et al., 2008). Here, the GA_3_ content change trends in infected and uninfected leaves were completely contrary. The GA_3_ content decreased continuously in uninfected leaves, whereas the content in infected leaves increased rapidly after the formation of disease spots, indicating that the GA_3_ content was directly induced by *S. sclerotiorum*. The GA_3_ content in infected areas increased without changes in the expression levels of GA-signaling components from 0 to 24 hpi, compared with the decreased content in uninfected areas (Figure 5B). These results suggest that, after a pathogen attack, plants increase the GA_3_ content in the infected area by transporting it from nearby areas rather than through early-stage biosynthesis.

The JA contents were lower in uninfected leaves, but the trend was consistent with previous reports (Pieterse et al., 2012; Wu et al., 2016). The transcriptomic data and qPCR results led us to speculate that the interactions between rapeseeds and *S. sclerotiorum* are complex. In this research, even though leaf necrosis was not shown, the gene encoding LOX2, which is a key enzyme in JA synthesis, was expressed at 6 hpi (Figures 1C, 4). Then, the upregulated expression levels of *JAR1, COI1*, and *MYC2* indicated that the JA-signaling pathway was activated at 6 hpi (Figure 3C). At the early stages of *S. sclerotiorum* infection, rapeseeds may induce a downstream defense response by synthesizing JA; consequently, numerous JA synthesis-related proteins should be expressed, and downstream signal transmission should be induced. However, although JA content dramatically increased at 24 hpi and 48 hpi, the symptoms in rapeseed leaves were more severe (Figures 1, 5D). At these times, *LOX2* and *AOS* expression levels were still increased, but the *JAR1, COI1*, and *MYC2* expression levels were decreased (Figures 3C, 4). The expression of the JAZ protein family increased, resulting in the inhibition of *MYC2* transcription factor activity, which was consistent with the findings of Wu et al. (2016). As the key protein of the JA-signaling pathway, JAZ degradation controls the activation of downstream genes (Chini et al., 2007). Bacteria and fungi secrete effectors or enzymes that prevent JAZ degradation in their host plants (Gimenez-Ibanez et al., 2014; Plett et al., 2014; Patkar et al., 2015; Dallery et al., 2020). Thus, the effectors from *S. sclerotiorum* may stabilize the JAZ proteins in rapeseeds. Although the JA content in the inoculated area was significantly increased at 24 and 48 hpi, the JA-responsive genes were repressed and failed in pathogen defense (Figures 1D, 5D). This study revealed that *S. sclerotiorum* failed to prevent JA synthesis in rapeseeds but successfully inhibited the JA-signaling pathway through unknown secreted proteins, resulting in the failure of related defense responses in rapeseeds. Future investigations will identify the related secreted proteins and their mechanisms.

Ethylene plays a positive role in the SSR resistance in *B. napus* (Yang et al., 2010; Wu et al., 2016). The ET content was not measured in this study, but the upregulated expression levels of *EIN3* and *ERF1/2* after 24 hpi indicated that the ET-signaling pathway was activated, which suggests that this pathway functions more in late-stage pathogen defenses (Figures 3A, 4).

Salicylic acid and its signaling pathway trigger resistance against biotrophic and hemibiotrophic pathogens (Glazebrook, 2005). Although the pathogen *S. sclerotiorum* is a necrotrophic pathogen, some reports indicate that SA positively regulates *S. sclerotiorum* resistance (Novákov et al., 2014; Ding et al., 2020). Here, the SA contents in infected leaves dramatically increased after 24 hpi, with SA signaling-related genes having upregulated expression levels, especially the defense-related *PR1* genes (Zhang et al., 1999; Figures 3B, 5E). The qRT-PCR analyses of the expression levels of genes such as *ICS1, MPK4*, and *EDS5* at different stages after *S. sclerotiorum* inoculation into rapeseed leaves confirmed that the SA synthesis in rapeseeds did involve the branch acid pathways. The *BnICS1* expression level was regulated by plant hormone networks and participated in the activating of the SA-signaling pathway at the early infection stages (Peng et al., 2016). These results suggested that the SA pathway aids the response of rapeseeds to the infection by *S. sclerotiorum* at late stages.

### The Battle of Cell Walls Both in *B. napus* and *S. sclerotiorum*

Once the fungus is established, a transition to necrotrophy occurs and host cell death pathways are subverted, inducing apoptotic cell death. This fungal-induced cell death provides nutrients that exclusively benefit the fungus. In pathogenic fungi, the cell wall plays a critical role during host invasion because it is the first structure to physically contact plant cells. It is then recognized by several plant components through microbe-associated molecular patterns to activate host immune responses (Latgé and Beauvais, 2014). During the invasion process*, S. sclerotiorum* secretes cell wall-degrading enzymes (CWDEs) to degrade the cell walls of the hosts, resulting in the death of plants cells. Meanwhile, the rapeseed continuously secretes hydrolytic enzymes, such as chitinases and glucanases, to degrade the cell walls of *S. sclerotiorum* (Stotz et al., 2011; Zhang et al., 2015). RNA-seq analysis of a global study on *S. sclerotiorum* gene expression, especially the genes encoding hydrolytic enzymes, transporters, and effectors as it infects *B. napus* from 0 to 48 hpi (Seifbarghi et al., 2017), found that many genes involved in polysaccharide degradation show high expression levels at 24 and 48 hpi, including *SSPG1* (SS1G_10167), *SSPG3* (SS1G_10698), and *exoPG1* (SS1G_04207). In our study, most genes encoding CWDEs in *S. sclerotiorum* were upregulated after 24 hpi and accompanied the appearance of leaf necrosis (Figures 1D, 7), which was consistent with the results of Seifbarghi et al. (2017). In our research, however, the expression level of *SSPG1* was downregulated at 6 hpi, which might be an effect of the partial resistance of rapeseed plants. Consequently, it is hypothesized that, during early-stage infections, *S. sclerotiorum* induces SA synthesis in rapeseeds to promote infection, and after the *S. sclerotiorum* infection is successful, numerous CWDEs are synthesized to ingest nutrients (Bashi et al., 2012).

In summary, the process of reciprocal evolution occurs as rapeseeds interact with *S. sclerotiorum*. Although rapeseeds have developed a complex defense system or acquired disease-resistance genes against diverse pathogens, it is difficult to avoid *S*. *sclerotiorum* infections, which can interfere with hormone synthesis and signaling pathways. Thus, analyzing the molecular mechanisms of the hormone-regulated metabolic networks involved in *B. napus*–*S. sclerotiorum* interactions and excavating related protein-protein interactions will aid in breeding *B. napus*. These results lay a foundation and provide new insights for further research.

## Data Availability Statement

The original contributions presented in the study are publicly available. This data can be found here: National Center for Biotechnology Information (NCBI) BioProject database under accession number PRJNA735329.

## Author Contributions

QP conceived and designed the experiments and amended the manuscript. JZ provided the rapeseed cultivars RS-1 and Ss1980, cultivated the plant samples, and inoculated the pathogens. SC and MH contributed to RNA extraction and qRT-PCR. BX and XG contributed to the measurement of hormones and data analysis and wrote the manuscript. All authors contributed to the article and approved the submitted version.

## Funding

This study was supported by the National Natural Science Foundation of China (No. 31771834), Natural Science Foundation of Jiangsu Province (No. BK20191237), and China Agriculture Research System of MOF and MARA (CARS-12).

## Conflict of Interest

The authors declare that the research was conducted in the absence of any commercial or financial relationships that could be construed as a potential conflict of interest. The reviewer X-LT declared a shared affiliation, with no collaboration, with one of the authors QP to the handling editor at the time of the review.

## Publisher's Note

All claims expressed in this article are solely those of the authors and do not necessarily represent those of their affiliated organizations, or those of the publisher, the editors and the reviewers. Any product that may be evaluated in this article, or claim that may be made by its manufacturer, is not guaranteed or endorsed by the publisher.
