# Supplementary material for: Transcriptome Analysis Reveals the Complex Molecular Mechanisms of Brassica napus–Sclerotinia sclerotiorum Interactions
Source: Front Plant Sci. 2021 Oct 8;12:716935. doi: 10.3389/fpls.2021.716935 (PMC8531588; doi:10.3389/fpls.2021.716935)
Supplement: Supplementary file 1 [file Table_1.DOCX]

Table S1 Primers used in this study.

| Name | Gene ID | Forward sequence (5’-3’) | Reverse sequence (5’-3’) |
| --- | --- | --- | --- |
| *Actin 2* | BnaC03g73810D | ACTCTCCAGCTATGTATGTCGCC | GAGACACACCATCACCAGAATCC |
| *UBC10* | BnaA10g06670D | GTTCTTCTTCTTCTCCGC | GGCATACTTCTGAGTCCAG |
| *YLS8* | BnaC09g47620D | AGAGACGAAACGATGTCG | AAGCCACAAGTTCACAGG |
| *PGIP1* | BnaA05g26870D | ATGCCGATGCTTGAGAC | CAAAGGAGCCGAATGAG |
| *PGIP2* | BnaA10g24090D | GAGTCATTTGGGTCGTTTC | TCCCTGTGATTCCGTTG |
| *MYC2* | BnaA05g18020D | ACAAGGCAAAGCCCAGACA | CTTCGCCCGCTCACAAC |
| *JAZ3* | BnaC05g35610D | AAACTACCATCTGCCTCACT | GAACATCCTGGCTCCCT |
| *JAZ1* | BnaC08g36840D | TTGGCGAGCAAAGGAAC | TTGGGATAGGAGCGATGT |
| *NPR1* | BnaC01g18080D | CGATAAGATTGCGGACTGT | AGAGGAACCTGACCGAGAC |
| *PR1* | BnaC03g45470D | CACGACAGGCAGTAGGC | TGACCCAAAGGTTCACG |
| *MPK4* | BnaC03g31910D | ACGCTCTAACCAACCCTT | TACAACCGACGGACCAG |
| *EDS5* | BnaC01g00120D | TTGCCAATGACAGTATCCC | AACCATATCCGCTTCGTG |
| *TGA3* | BnaA09g30910D | AGCTGATGCTGCAAAGG | CTCGGAAGGGCGGAAAC |
| *COI1* | BnaC03g22310D | TCTGCCACTAGACAACGG | AGAAGCATCCACCTCACG |
| *PDF1.2* | BnaA07g32150D | TTCGCTGCTCTTGTTGTT | CAGGCGTTATTGTTTCCA |
| *AOS* | BnaC02g29610D | TCCTTCACCTCCTTTCCC | AAGTAATCGTTGCGGTCTTT |
| *LOX2* | BnaA07g19600D | GCAAGACTGGTCGCAAAT | ATGCCGTTCCCTTGGTT |
| *ERF1-1* | BnaA06g30550D | TTTGAGGAGATAAGCCAGGAC | TACTCGTTAGCCAGCCACT |
| *EIN2* | BnaA10g26670D | ACCGCTGTTCCGCATTG | AAACCGCCAGCCCAGTC |
| *JAR1* | BnaC04g01170D | AATGACGAGAAACGCTGAC | GCAAGTAAATGGCGGATT |
| *GERMIN* | BnaA07g30320D | TGCGTAGCCAACCTCAAA | TGAATCCAGCGGTGATAGAG |
| *ICS* | BnaC06g22820D | AAGTCGCCTCTGATTCGC | AGGGCTCCCACTCCACA |
| *WRKY70* | BnaC08g27340D | CTCAAGACGGGTCGGATTT | CGTTGTCGCAGGAAGCA |
| *WRKY33* | BnaA03g17820D | ATGAGTTTGAACGAGGCT | CCATTACCATTCCCACC |
| *WRKY29* | BnaA03g46020D | ATGGGTGAGGTGGCTTAT | CTCAAATGGGAGGCAAG |
