# Supplementary material for: Transcriptome Analysis Reveals the Complex Molecular Mechanisms of Brassica napus–Sclerotinia sclerotiorum Interactions
Source: Front Plant Sci. 2021 Oct 8;12:716935. doi: 10.3389/fpls.2021.716935 (PMC8531588; doi:10.3389/fpls.2021.716935)
Supplement: Supplementary file 2 [file Table_2.DOCX]

Table S2 Sequencing and assembly statistics for the 12 transcriptomes data of *B. napus* at 3 stages after incubation and mock.

|  | Total Reads | Clean Reads | Mapped Reads | Uniq Mapped Reads | Multiple Map Reads |
| --- | --- | --- | --- | --- | --- |
| 0hpi-1 | 53,594,478 | 26,797,239 | 15,325,271 | 13,637,254 | 1,688,017 |
| 0hpi-2 | 53,272,928 | 26,636,464 | 16,857,788 | 14,909,470 | 1,948,318 |
| 0hpi-3 | 66,475,206 | 33,237,603 | 20,618,794 | 18,247,686 | 2,371,108 |
| 6hpi-1 | 47,407,572 | 23,703,786 | 32,237,383 | 27,744,350 | 4,493,033 |
| 6hpi-2 | 44,753,524 | 22,376,762 | 32,086,014 | 28,020,796 | 4,065,218 |
| 6hpi-3 | 55,946,226 | 27,973,113 | 39,283,631 | 34,436,584 | 4,847,047 |
| 24hpi-1 | 53,740,962 | 26,870,481 | 38,915,183 | 32,346,889 | 6,568,294 |
| 24hpi-2 | 57,373,582 | 28,686,791 | 40,398,679 | 33,763,752 | 6,634,927 |
| 24hpi-3 | 52,476,790 | 26,238,395 | 34,978,889 | 30,221,452 | 4,757,437 |
| 48hpi-1 | 51,552,226 | 25,776,113 | 29,303,198 | 26,299,016 | 3,004,182 |
| 48hpi-2 | 45,323,556 | 22,661,778 | 27,211,726 | 24,097,586 | 3,114,140 |
| 48hpi-3 | 47,198,054 | 23,599,027 | 31,641,950 | 26,488,057 | 5,153,893 |
