# Supplementary material for: Transcriptome Analysis Reveals the Complex Molecular Mechanisms of Brassica napus–Sclerotinia sclerotiorum Interactions
Source: Front Plant Sci. 2021 Oct 8;12:716935. doi: 10.3389/fpls.2021.716935 (PMC8531588; doi:10.3389/fpls.2021.716935)
Supplement: Supplementary file 4 [file Table_4.DOCX]

Table S4 The GO enrichment of all different expression genes in *B.napus*.

| Term | Ontology | Items number | P-value |
| --- | --- | --- | --- |
| 1.GO enrichment of genes clustered in Group 1 | | | |
| Response to oxidative stress | Biological Process | 182 | 0 |
| Regulation of hydrogen peroxide metabolic process | Biological Process | 200 | 0 |
| Defense response to fungus, incompatible interaction | Biological Process | 60 | 0 |
| Toxin catabolic process | Biological Process | 249 | 0 |
| Systemic acquired resistance | Biological Process | 301 | 0 |
| Response to heat | Biological Process | 180 | 0 |
| Response to jasmonic acid | Biological Process | 223 | 0 |
| Negative regulation of programmed cell death | Biological Process | 334 | 0 |
| Proline transport | Biological Process | 87 | 0 |
| Defense response by callose deposition | Biological Process | 110 | 0 |
| Protein targeting to membrane | Biological Process | 556 | 0 |
| Response to salt stress | Biological Process | 489 | 0 |
| Para-aminobenzoic acid metabolic process | Biological Process | 89 | 0 |
| Response to endoplasmic reticulum stress | Biological Process | 248 | 0 |
| Endoplasmic reticulum unfolded protein response | Biological Process | 386 | 0 |
| Response to sucrose | Biological Process | 187 | 0 |
| Hyperosmotic salinity response | Biological Process | 256 | 0 |
| Regulation of plant-type hypersensitive response | Biological Process | 559 | 0 |
| Coumarin biosynthetic process | Biological Process | 104 | 0 |
| RNA methylation | Biological Process | 315 | 0 |
| 2.GO enrichment of genes clustered in Group 2 | | | |
| Cuticle development | Biological Process | 88 | 0 |
| Pattern specification process | Biological Process | 84 | 0 |
| Auxin polar transport | Biological Process | 104 | 0 |
| Photosynthesis | Biological Process | 92 | 0 |
| Transmembrane receptor protein tyrosine kinase signaling pathway | Biological Process | 121 | 0 |
| Regulation of hormone levels | Biological Process | 80 | 0 |
| Polysaccharide biosynthetic process | Biological Process | 143 | 0 |
| Defense response to bacterium | Biological Process | 369 | 0 |
| Glycine catabolic process | Biological Process | 90 | 0 |
| Regulation of cell size | Biological Process | 93 | 0 |
| Photosynthesis, light reaction | Biological Process | 133 | 0 |
| Chlorophyll catabolic process | Biological Process | 70 | 0 |
| Unsaturated fatty acid biosynthetic process | Biological Process | 148 | 0 |
| Microtubule nucleation | Biological Process | 105 | 0 |
| Plant-type cell wall | Cellular Component | 256 | 0 |
| Anchored component of plasma membrane | Cellular Component | 93 | 0 |
| Integral component of membrane | Cellular Component | 833 | 0 |
| Chloroplast envelope | Cellular Component | 839 | 0 |
| Thylakoid | Cellular Component | 104 | 0 |
| Apoplast | Cellular Component | 408 | 0 |
