# Supplementary material for: Transcriptome Analysis Reveals the Complex Molecular Mechanisms of Brassica napus–Sclerotinia sclerotiorum Interactions
Source: Front Plant Sci. 2021 Oct 8;12:716935. doi: 10.3389/fpls.2021.716935 (PMC8531588; doi:10.3389/fpls.2021.716935)
Supplement: Supplementary file 5 [file Table_5.DOCX]

Table S5 KEGG analysis of different expression genes in *B. napus* at 6-, 24- and 48-hpi

| #Pathway | KO | Enrichment_  Factor | Q-value | Gene  Number |
| --- | --- | --- | --- | --- |
| 1.KEGG enrichment of DEGs at 6hpi | | | | |
| Circadian rhythm - plant | ko04712 | 6.52 | <0.01 | 19 |
| Tryptophan metabolism | ko00380 | 4.84 | <0.01 | 15 |
| Butanoate metabolism | ko00650 | 5.45 | <0.01 | 9 |
| Starch and sucrose metabolism | ko00500 | 2.05 | <0.01 | 34 |
| Carotenoid biosynthesis | ko00906 | 4.25 | <0.01 | 10 |
| Sulfur metabolism | ko00920 | 3.62 | 0.02 | 11 |
| Histidine metabolism | ko00340 | 4.56 | 0.04 | 8 |
| Sesquiterpenoid and triterpenoid biosynthesis | ko00909 | 5.48 | 0.07 | 6 |
| Limonene and pinene degradation | ko00903 | 3.36 | 0.08 | 10 |
| Cyanoamino acid metabolism | ko00460 | 2.48 | 0.16 | 14 |
| Ribosome biogenesis in eukaryotes | ko03008 | 2.17 | 0.23 | 17 |
| Valine, leucine and isoleucine degradation | ko00280 | 2.83 | 0.29 | 10 |
| Glyoxylate and dicarboxylate metabolism | ko00630 | 2.33 | 0.30 | 14 |
| Carbon fixation in photosynthetic organisms | ko00710 | 2.31 | 0.33 | 14 |
| beta-Alanine metabolism | ko00410 | 2.75 | 0.36 | 10 |
| Pentose and glucuronate interconversions | ko00040 | 2.13 | 0.37 | 16 |
| Tropane, piperidine and pyridine alkaloid biosynthesis | ko00960 | 2.83 | 0.47 | 9 |
| Synthesis and degradation of ketone bodies | ko00072 | 8.07 | 0.56 | 3 |
| Biosynthesis of unsaturated fatty acids | ko01040 | 2.69 | 0.65 | 9 |
| Ascorbate and aldarate metabolism | ko00053 | 2.81 | 0.78 |  |
| 2.KEGG enrichment of DEGs at 24hpi | | | | |
| Ribosome | ko03010 | 2.21 | 0 | 90 |
| Glutathione metabolism | ko00480 | 4.08 | 0 | 44 |
| Ribosome biogenesis in eukaryotes | ko03008 | 3.30 | <0.01 | 37 |
| Biosynthesis of amino acids | ko01230 | 1.97 | <0.01 | 59 |
| alpha-Linolenic acid metabolism | ko00592 | 3.57 | <0.01 | 17 |
| Stilbenoid, diarylheptanoid and gingerol biosynthesis | ko00945 | 3.58 | <0.01 | 16 |
| Sulfur metabolism | ko00920 | 3.45 | <0.01 | 15 |
| Limonene and pinene degradation | ko00903 | 3.05 | 0.04 | 13 |
| Vitamin B6 metabolism | ko00750 | 4.95 | 0.12 | 6 |
| Pentose phosphate pathway | ko00030 | 2.32 | 0.31 | 14 |
| Phenylalanine, tyrosine and tryptophan biosynthesis | ko00400 | 2.22 | 0.62 | 13 |
| ABC transporters | ko02010 | 2.88 | 0.67 | 8 |
| Cysteine and methionine metabolism | ko00270 | 1.80 | 0.84 | 20 |
| Degradation of aromatic compounds | ko01220 | 4.36 | 1 | 4 |
| Indole alkaloid biosynthesis | ko00901 | 9.66 | 1 | 2 |
| Glycolysis / Gluconeogenesis | ko00010 | 1.42 | 1 | 19 |
| Carbon metabolism | ko01200 | 1.25 | 1 | 36 |
| Lysine biosynthesis | ko00300 | 2.25 | 1 | 4 |
| Glucosinolate biosynthesis | ko00966 | 3.56 | 1 | 2 |
| Fructose and mannose metabolism | ko00051 | 1.53 | 1 | 11 |
| 3.KEGG enrichment of DEGs at 48hpi | | | | |
| Carbon metabolism | ko01200 | 1.71 | 0 | 348 |
| Biosynthesis of amino acids | ko01230 | 1.53 | 0 | 326 |
| Glutathione metabolism | ko00480 | 1.83 | 0 | 140 |
| Ribosome | ko03010 | 1.68 | 0 | 485 |
| Carbon fixation in photosynthetic organisms | ko00710 | 2.18 | 0 | 134 |
| Glyoxylate and dicarboxylate metabolism | ko00630 | 1.94 | 0 | 118 |
| Photosynthesis - antenna proteins | ko00196 | 4.08 | <0.01 | 65 |
| Photosynthesis | ko00195 | 2.53 | <0.01 | 134 |
| Pentose phosphate pathway | ko00030 | 1.99 | <0.01 | 85 |
| Porphyrin and chlorophyll metabolism | ko00860 | 1.88 | <0.01 | 63 |
| Phenylalanine, tyrosine and tryptophan biosynthesis | ko00400 | 1.76 | <0.01 | 73 |
| Glycine, serine and threonine metabolism | ko00260 | 1.59 | <0.01 | 99 |
| Sulfur metabolism | ko00920 | 1.85 | <0.01 | 57 |
| Nitrogen metabolism | ko00910 | 1.83 | <0.01 | 56 |
| Carotenoid biosynthesis | ko00906 | 1.88 | <0.01 | 45 |
| Glycolysis / Gluconeogenesis | ko00010 | 1.40 | <0.01 | 132 |
| Biosynthesis of unsaturated fatty acids | ko01040 | 1.68 | <0.01 | 57 |
| 2-Oxocarboxylic acid metabolism | ko01210 | 1.49 | <0.01 | 89 |
| Ascorbate and aldarate metabolism | ko00053 | 1.66 | <0.01 | 48 |
| Citrate cycle (TCA cycle) | ko00020 | 1.47 | <0.01 | 78 |
