# Supplementary material for: Transcriptome Analysis Reveals the Complex Molecular Mechanisms of Brassica napus–Sclerotinia sclerotiorum Interactions
Source: Front Plant Sci. 2021 Oct 8;12:716935. doi: 10.3389/fpls.2021.716935 (PMC8531588; doi:10.3389/fpls.2021.716935)
Supplement: Supplementary file 8 [file Table_8.DOCX]

Table S8 KEGG analysis of different expression genes in *S. sclerotiorum* at 6-, 24- and 48-hpi

| #Pathway | KO | Enrichment_  Factor | Q-value | Gene  Number |
| --- | --- | --- | --- | --- |
| 1.KEGG enrichment of DEGs at 6hpi | | | | |
| Ribosome | ko03010 | 4.92 | <0.01 | 84 |
| Ribosome biogenesis in eukaryotes | ko03008 | 2.51 | <0.01 | 28 |
| Purine metabolism | ko00230 | 1.60 | 1 | 22 |
| Pyrimidine metabolism | ko00240 | 1.52 | 1 | 16 |
| RNA polymerase | ko03020 | 1.81 | 1 | 8 |
| Caffeine metabolism | ko00232 | 4.06 | 1 | 2 |
| Taurine and hypotaurine metabolism | ko00430 | 2.22 | 1 | 4 |
| Vitamin B6 metabolism | ko00750 | 2.61 | 1 | 3 |
| Other glycan degradation | ko00511 | 3.05 | 1 | 2 |
| Tryptophan metabolism | ko00380 | 1.52 | 1 | 8 |
| Phenylalanine metabolism | ko00360 | 1.69 | 1 | 5 |
| Glyoxylate and dicarboxylate metabolism | ko00630 | 1.47 | 1 | 7 |
| Valine, leucine and isoleucine biosynthesis | ko00290 | 1.74 | 1 | 4 |
| One carbon pool by folate | ko00670 | 1.63 | 1 | 4 |
| Tyrosine metabolism | ko00350 | 1.45 | 1 | 5 |
| Biosynthesis of unsaturated fatty acids | ko01040 | 1.52 | 1 | 4 |
| Starch and sucrose metabolism | ko00500 | 1.24 | 1 | 10 |
| 2.KEGG enrichment of DEGs at 24hpi | | | | |
| Valine, leucine and isoleucine degradation | ko00280 | 3.61 | <0.01 | 13 |
| Pentose and glucuronate interconversions | ko00040 | 3.99 | <0.01 | 11 |
| Starch and sucrose metabolism | ko00500 | 2.55 | 0.03 | 15 |
| Peroxisome | ko04146 | 2.33 | 0.15 | 14 |
| Biosynthesis of amino acids | ko01230 | 1.71 | 0.52 | 23 |
| Taurine and hypotaurine metabolism | ko00430 | 3.79 | 0.58 | 5 |
| Fructose and mannose metabolism | ko00051 | 2.67 | 0.65 | 8 |
| Tyrosine metabolism | ko00350 | 2.78 | 0.84 | 7 |
| Amino sugar and nucleotide sugar metabolism | ko00520 | 1.96 | 1 | 12 |
| Aminoacyl-tRNA biosynthesis | ko00970 | 2.03 | 1 | 10 |
| Glycine, serine and threonine metabolism | ko00260 | 2.03 | 1 | 9 |
| Tryptophan metabolism | ko00380 | 2.08 | 1 | 8 |
| Degradation of aromatic compounds | ko01220 | 2.38 | 1 | 6 |
| Carbon metabolism | ko01200 | 1.56 | 1 | 18 |
| Caffeine metabolism | ko00232 | 5.56 | 1 | 2 |
| Vitamin B6 metabolism | ko00750 | 3.57 | 1 | 3 |
| Propanoate metabolism | ko00640 | 2.45 | 1 | 5 |
| Butanoate metabolism | ko00650 | 2.45 | 1 | 5 |
| Glyoxylate and dicarboxylate metabolism | ko00630 | 2.01 | 1 | 7 |
| Phenylalanine metabolism | ko00360 | 2.32 | 1 | 5 |
| 3.KEGG enrichment of DEGs at 48hpi | | | | |
| Pentose and glucuronate interconversions | ko00040 | 4.21 | <0.01 | 14 |
| Starch and sucrose metabolism | ko00500 | 2.54 | <0.01 | 18 |
| Degradation of aromatic compounds | ko01220 | 3.29 | 0.02 | 10 |
| Amino sugar and nucleotide sugar metabolism | ko00520 | 2.30 | 0.04 | 17 |
| Ribosome | ko03010 | 1.79 | 0.10 | 27 |
| Cyanoamino acid metabolism | ko00460 | 2.76 | 0.45 | 8 |
| Arginine and proline metabolism | ko00330 | 1.95 | 1 | 11 |
| Glycosaminoglycan degradation | ko00531 | 6.91 | 1 | 2 |
| alpha-Linolenic acid metabolism | ko00592 | 4.15 | 1 | 3 |
| Arachidonic acid metabolism | ko00590 | 4.15 | 1 | 3 |
| Ascorbate and aldarate metabolism | ko00053 | 4.15 | 1 | 3 |
| Glycerolipid metabolism | ko00561 | 2.13 | 1 | 8 |
| Propanoate metabolism | ko00640 | 2.44 | 1 | 6 |
| Various types of N-glycan biosynthesis | ko00513 | 2.05 | 1 | 8 |
| Peroxisome | ko04146 | 1.66 | 1 | 12 |
| Carotenoid biosynthesis | ko00906 | 4.61 | 1 | 2 |
| Fatty acid metabolism | ko01212 | 1.84 | 1 | 8 |
| Taurine and hypotaurine metabolism | ko00430 | 2.51 | 1 | 4 |
| Nitrogen metabolism | ko00910 | 2.03 | 1 | 5 |
| Other glycan degradation | ko00511 | 3.45 | 1 | 2 |
